# Is fever control or improved survival the ‘risk factor’ for ventilator-associated pneumonia?

**DOI:** 10.1186/s13054-015-0821-0

**Published:** 2015-04-30

**Authors:** Matthew BA Harmon, Theis S Itenov, Jens U Jensen, Nicole P Juffermans

**Affiliations:** Department of Intensive Care Medicine, Laboratory of Experimental Intensive Care and Anesthesiology, Academic Medical Center, University of Amsterdam, Meibergdreef 9, Amsterdam, 1105 AZ The Netherlands; Nordsjællands Hospital, Department of Anesthesiology, Hillerød, Denmark; Department of Rheumatology and Infectious Diseases, Rigshospitalet, University of Copenhagen, CHIP, Finsen Centret, Copenhagen, Denmark

We read with interest the paper by Launey and colleagues [1] regarding the effect of fever control on the incidence of ventilator-associated pneumonia (VAP) in brain-injured patients. We commend the authors for addressing this relationship, given that fever and nosocomial infections are frequent in these patients. However, we have concerns regarding the interpretation of data.

Firstly, independent risk factors for VAP, namely disease severity and lung contusion [2,3], are unevenly distributed between groups. In addition, other risk factors, such as chronic obstructive pulmonary disease (COPD) and ICU readmission [2,4], were not taken into account, suggesting any effect of fever control is likely subject to confounding.

Secondly, mortality in the control group was higher (34% versus 23%), lowering the observation period if death occurred within 28 days. Thus, patients in the fever-control group were observed for a longer period of time, increasing their time at risk for VAP.

Thirdly, VAP incidence was higher in patients who were subject to fever control for longer than 3 days. However, duration of fever control was likely determined by factors also affecting the patients’ risk of acquiring VAP; that is, death. Not surprisingly, those who died within 3 or fewer days of initiating fever control did not develop VAP as frequently as those who did not.

In summary, the intervention group and historical control group in this study do not seem to be optimally matched on crucial parameters. Additionally, decreased mortality and longer follow-up in the intervention group likely resulted in uncontrolled lead time bias/attrition bias. Decreased mortality also seems contradictory to the claim that VAP, a deadly condition for ICU patients, is more frequent among fever-control patients.

## Abbreviations

COPD: Chronic obstructive pulmonary disease
VAP: Ventilator-associated pneumonia
