# Supplementary material for: Is fever control or improved survival the ‘risk factor’ for ventilator-associated pneumonia?
Source: Crit Care. 2015 Apr 30;19(1):208. doi: 10.1186/s13054-015-0821-0 (PMC4415296; doi:10.1186/s13054-015-0821-0)
Supplement: Related texts

# Reply: Author response

**Authors:** Yoann Launey, Nicolas Nesseler, Fanny Feuillet, Yannick Mallédant, Philippe Seguin

We thanks Dr Harmon and colleagues for their comments, which we would like to nuance.

Indeed, COPD was not reported at admission; however, such disease is clearly unusual in the studied population. In a recent multicentre study performed in the same population, a history of respiratory disease (including COPD) was observed in only 3.6% [5]. Secondly, none of the included patients were readmitted in our ICU during the first 28 days of ICU stay, which was the endpoint period to assess VAP occurrence.

As reported in the statistical section, we used a competing risk multivariate model [6] which considers death within 28 days as a competing event. Consequently, this model takes into account only the periods of time during which the patients were exposed to the risk of VAP. Moreover, the benefit of this model is that the death event is not a confounding factor in the determination of VAP incidence between the patients receiving fever control for longer than 3 days and those who did not. The size of the study was not large enough to reach statistical significance.

Finally, we acknowledge that the matching of the two groups was not optimal in our observational study and obviously a prospective randomized study is required. Nevertheless, we would like to emphasize our study was not designed to assess the impact of fever-control strategy on mortality and, despite the apparent results, no conclusion can be drawn on mortality. Moreover, a recent meta-analysis showed VAP was not associated with over-mortality in medical and trauma patients [7].
